# Alterations in cellular and organellar phospholipid compositions of HepG2 cells during cell growth

**DOI:** 10.1038/s41598-021-81733-3

**Published:** 2021-02-01

**Authors:** Tokuji Tsuji, Shin-ya Morita, Yoshinobu Nakamura, Yoshito Ikeda, Taiho Kambe, Tomohiro Terada

**Affiliations:** 1 Department of Pharmacy, Shiga University of Medical Science Hospital, Otsu, Shiga 520-2192 Japan; 2 Graduate School of Biostudies, Kyoto University, Kyoto, 606-8502 Japan

**Keywords:** Membrane lipids, Phospholipids

## Abstract

The human hepatoblastoma cell line, HepG2, has been used for investigating a wide variety of physiological and pathophysiological processes. However, less information is available about the phospholipid metabolism in HepG2 cells. In the present report, to clarify the relationship between cell growth and phospholipid metabolism in HepG2 cells, we examined the phospholipid class compositions of the cells and their intracellular organelles by using enzymatic fluorometric methods. In HepG2 cells, the ratios of all phospholipid classes, but not the ratio of cholesterol, markedly changed with cell growth. Of note, depending on cell growth, the phosphatidic acid (PA) ratio increased and phosphatidylcholine (PC) ratio decreased in the nuclear membranes, the sphingomyelin (SM) ratio increased in the microsomal membranes, and the phosphatidylethanolamine (PE) ratio increased and the phosphatidylserine (PS) ratio decreased in the mitochondrial membranes. Moreover, the mRNA expression levels of enzymes related to PC, PE, PS, PA, SM and cardiolipin syntheses changed during cell growth. We suggest that the phospholipid class compositions of organellar membranes are tightly regulated by cell growth. These findings provide a basis for future investigations of cancer cell growth and lipid metabolism.

## Introduction

Liver cancer is the seventh most commonly diagnosed cancer and the third leading cause of cancer death worldwide, and mainly includes hepatocellular carcinoma, cholangiocarcinoma and hepatoblastoma^1,2^. Hepatoblastoma is the most common malignant hepatic tumor in pediatrics and is considered to originate from hepatoblasts or embryonic liver progenitor cells^3^. The HepG2 cell line was originally isolated from primary hepatoblastoma of a 15-year-old Caucasian male, and exhibits epithelial-like morphology^4–6^. In addition to cancer studies, HepG2 cells have been widely used as an in vitro liver model for lipid and lipoprotein metabolism, bile salt metabolism, drug metabolism, toxicity, biliary excretion, insulin resistance and infection studies^5,7–15^.

In recent years, there is growing evidence for a relationship between hepatic dysfunction and alterations in membrane phospholipid compositions^16,17^. Phospholipids are essential components of bilayer membranes in mammalian cells, and well known to be involved in numerous cellular processes, including cell growth, mitochondrial energy production, apoptosis, membrane trafficking, autophagy and intracellular signaling^18–23^. Phospholipids are divided into glycerophospholipids and sphingophospholipids based on their backbone structures. Depending on the head group structures, glycerophospholipids are further classified into phosphatidylcholine (PC), phosphatidylethanolamine (PE), phosphatidylserine (PS), phosphatidic acid (PA), phosphatidylinositol (PI), phosphatidylglycerol (PG) and cardiolipin (CL)^24^. Sphingophospholipids have a sphingosine backbone, and sphingomyelin (SM) is the most predominant sphingophospholipid in mammalian cells and plasma lipoproteins^24^. In mammalian cell membranes, cholesterol is an essential regulator of membrane physical properties such as fluidity^25^.

In mammalian cells, biosynthesis of phospholipids mainly occurs at the endoplasmic reticulum (ER), Golgi apparatus and mitochondria via the actions of numerous enzymes (Supplementary Fig. S1). The majority of hepatocellular PC molecules are synthesized from phosphocholine through the CDP-choline pathway, including CTP:phosphocholine cytidylyltransferase (CCT) and CDP-choline:diacylglycerol cholinephosphotransferase (CPT)^26^. In addition to the CDP-choline pathway, PE *N*-methyltransferase (PEMT) catalyzes the methylation of PE and produces approximately 30% of PC in liver cells^15,26^. In the CDP-ethanolamine pathway for PE biosynthesis, a sequence of reactions catalyzed by CTP:phosphoethanolamine cytidylyltransferase (ECT) and CDP-ethanolamine:diacylglycerol ethanolaminephosphotransferase (EPT) produces PE from phosphoethanolamine^26^. PS decarboxylase (PSD) is an enzyme to synthesize PE from PS in mitochondria^26,27^. PS synthase 1 (PSS1) and 2 (PSS2) are responsible for the exchange of l-serine with polar head groups of PC and PE, respectively^26,28^. Diacylglycerol kinase (DGK) catalyzes the phosphorylation of diacylglycerol (DG) to produce PA^18^. Phospholipase D1 (PLD1) and D2 (PLD2) are enzymes for PA production from PC^29,30^. PA is an essential substrate of CDP-DG synthase 1 (CDS1) or 2 (CDS2) to generate CDP-DG, which is an intermediate to produce PI via PI synthase (PIS) in the ER^31,32^. CDP-DG is also necessary for PG and CL biosynthesis in the mitochondrial membrane via phosphatidylglycerophosphate synthase 1 (PGS1) and CL synthase (CLS), respectively^33–35^. Serine palmitoyltransferase (SPT) catalyzes the first committed step in ceramide synthesis. Lastly, SM is produced by the transfer of phosphocholine from PC to ceramide, which predominantly occurs at the luminal side of trans-Golgi membranes by SM synthase 1 (SMS1) or at the plasma membranes by SM synthase 2 (SMS2)^36^.

The functions and morphology of HepG2 cells change with cell growth^5,9,37^. The phospholipid composition affects membrane shape, activity and localization of membrane proteins, cell growth and cell migration^18,21,38^. Therefore, in HepG2 cells, the changes in phospholipid compositions that occur during cell growth are predicted to affect cellular functions. However, the alterations of phospholipid class composition in HepG2 cells and their intracellular organelles during cell growth have not been investigated because of the difficulty in quantifying low levels of phospholipid classes in sparse cells and isolated intracellular organelles by using conventional methods. Recently, we have developed fluorometric assays for all major phospholipid classes including PC, PE, PS, PI, PA, PG, CL and SM, using combinations of specific enzymes and Amplex Red, which enable simple, sensitive (pico-molar range) and high-throughput quantification^24,39–44^. In the present study, we assessed the effects of cell growth on the phospholipid class compositions in HepG2 cells and their nuclei, microsomes and mitochondria using our enzymatic assays, and investigated the changes in mRNA expression of phospholipid synthesis-related enzymes during cell growth.

## Results and discussion

### Growth of HepG2 cells

**Figure 1:**
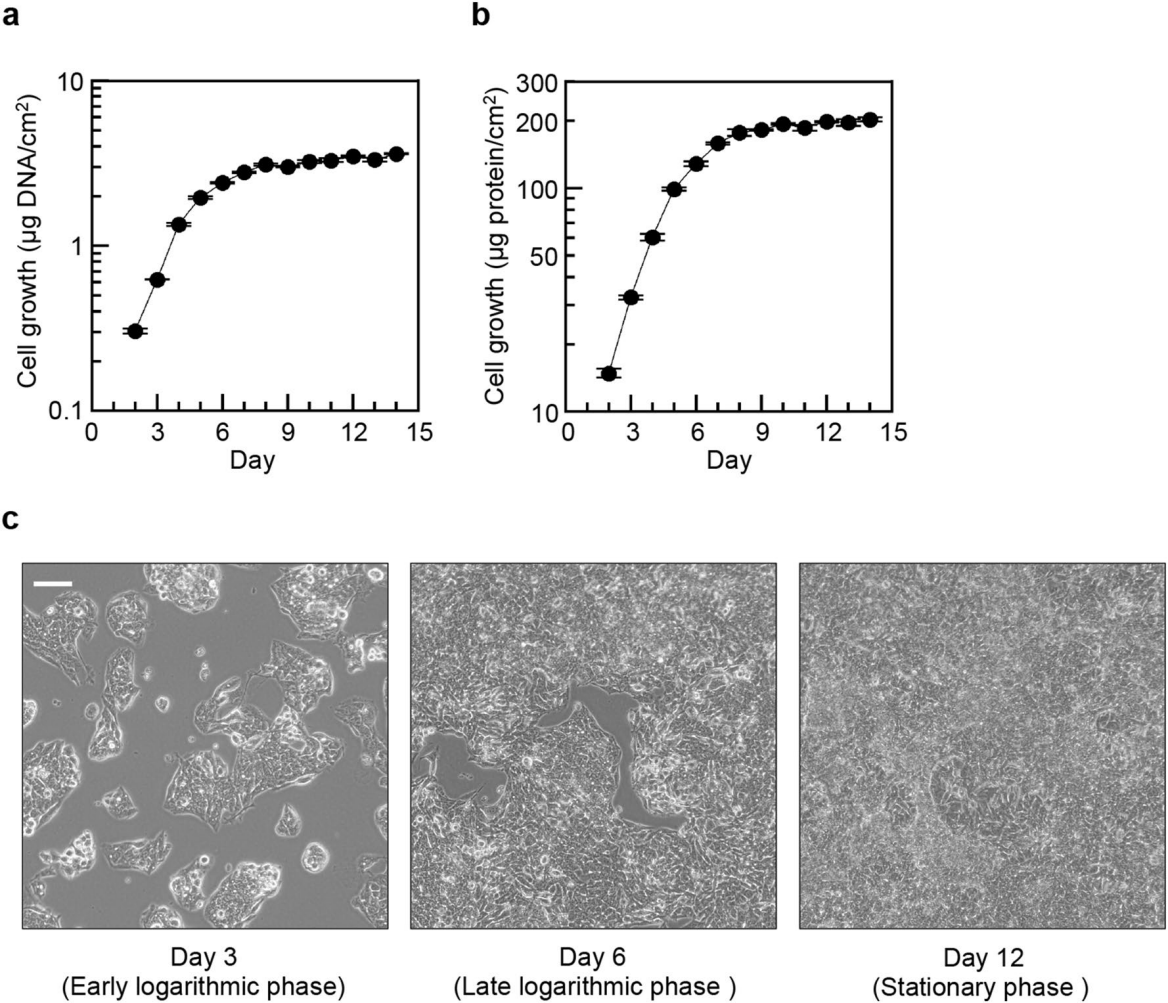
Cell growth of HepG2 cells. Cells were seeded at a density of 5.0 × 10^4^ cells/cm^2^ in six-well plates (Day 0). (**a**,**b**) Growth curves of HepG2 cells were determined by the measurement of total cellular DNA (**a**) and total cellular protein (**b**). Each point represents the mean value of three biologically independent experiments of duplicate measurements (mean ± S.E., n = 4). (**c**) Differential interference contrast images of HepG2 cells at Day 3 (early logarithmic phase), Day 6 (late logarithmic phase) and Day 12 (stationary phase). The scale bar represents 100 μm.

To observe cell growth, HepG2 cells seeded at a density of 5.0 × 10^4^ cells/cm^2^ were cultured in DMEM containing 10% FBS for up to 14 days. Cell growth was evaluated by measuring cellular DNA or protein. Growth curve analyses demonstrated that HepG2 cells grew in the logarithmic phase from Day 2 to Day 5 (Fig. 1a,b). Then, the cell growth rate gradually decreased, and the cells entered into stationary phase at Day 8 under the experimental conditions. Figure 1c shows images of HepG2 cells at Day 3 in the early logarithmic phase, at Day 6 in the late logarithmic phase and at Day 12 in the stationary phase. HepG2 cells grew as a monolayer at Day 3, whereas the cells were overconfluent as a multilayer at Day 12.

### Phospholipid composition in HepG2 cells in different growth phases

**Figure 2:**
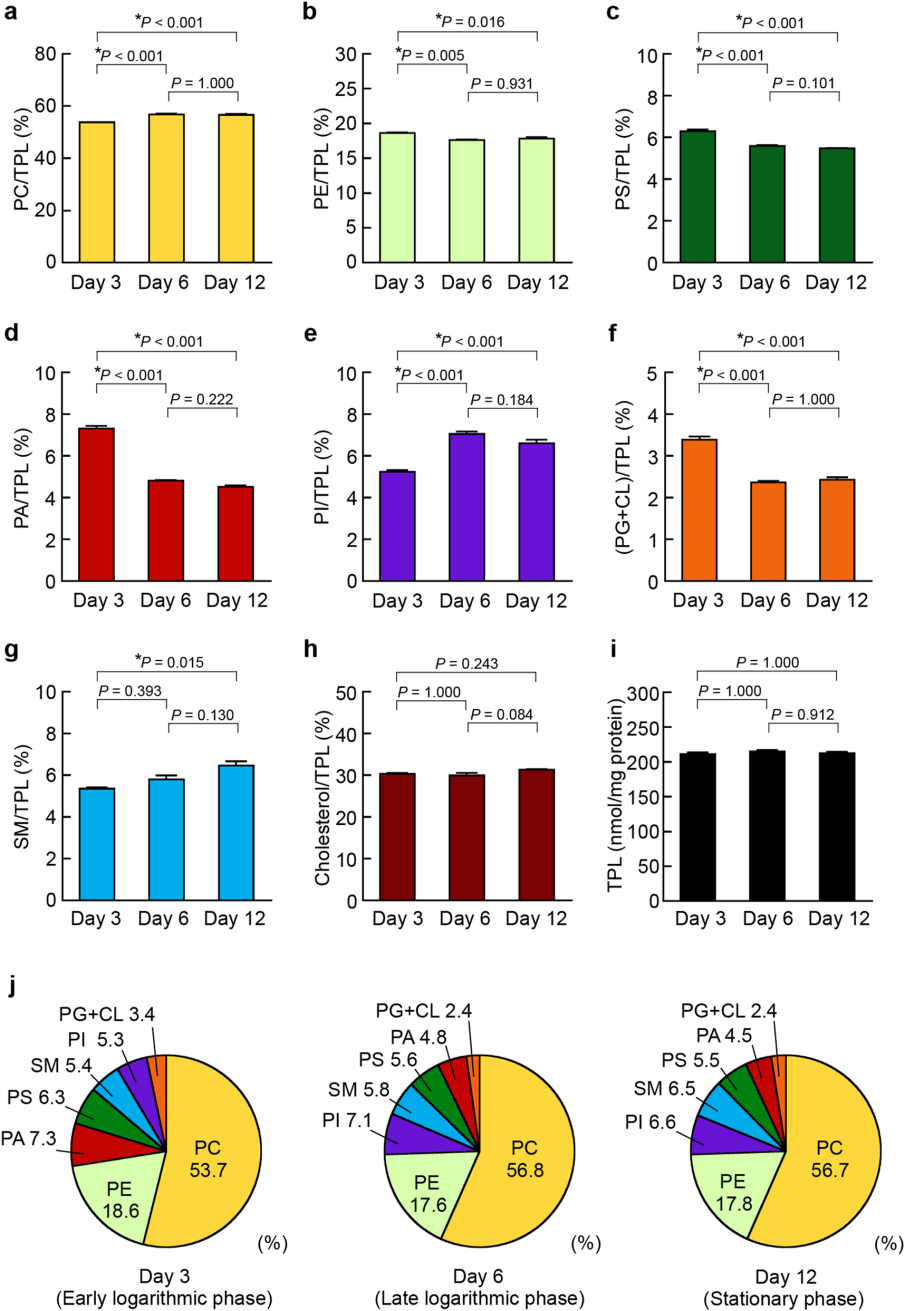
Alteration of phospholipid composition in HepG2 cells. HepG2 cells were seeded at a density of 5.0 × 10^4^ cells/cm^2^ in 75-cm^2^ flasks and cultured in DMEM containing 10% FBS at 37 °C for the indicated days. At Day 3 (early logarithmic phase), Day 6 (late logarithmic phase) and Day 12 (stationary phase), cellular lipids were extracted. The contents of PC, PE, PS, PA, PI, PG + CL, SM and cholesterol in HepG2 cells were determined by the enzymatic measurements and protein assay (see Supplementary Table S1). The ratios of PC/TPL (**a**), PE/TPL (**b**), PS/TPL (**c**), PA/TPL (**d**), PI/TPL (**e**), (PG + CL)/TPL (**f**), SM/TPL (**g**) and cholesterol/TPL (**h**) at Day 3, Day 6 and Day 12 were evaluated. The total phospholipid (TPL) content (**i**) was calculated as the sum of PC, PE, PS, PA, PI, PG + CL and SM contents (mean ± S.E., n = 3, *P < 0.05, significantly different among Day 3, Day 6 and Day 12, one-way ANOVA followed by the Bonferroni test). (**j**) The phospholipid compositions in HepG2 cells at Day 3, Day 6 and Day 12 are shown as pie charts.

The HepG2 cell line is one of the most widely used models for studying hepatic lipid metabolism^5,12^, whereas the basic information about the phospholipid compositions of the cellular membranes is scarce. Previously, we have found that the contents of phospholipid classes change in human embryonic kidney HEK293 cells depending on the cell density^44^. In the present study, we investigated whether the lipid contents in HepG2 cells were altered during cell growth. Using the enzymatic fluorometric methods that we developed previously, we examined the contents of all phospholipid classes and cholesterol in HepG2 cells at Day 3 in the early logarithmic phase, at Day 6 in the late logarithmic phase and at Day 12 in the stationary phase of cell growth (Supplementary Table S1). To evaluate changes in phospholipid class composition of whole cells, we determined the content ratio of each phospholipid class to total phospholipids (TPL). As a result, the cellular PC/TPL and PI/TPL ratios increased from Day 3 to Day 6, and the SM/TPL ratio at Day 12 was significantly higher than that at Day 3 (Fig. 2a,e,g). On the other hand, the PE/TPL, PS/TPL, PA/TPL and (PG + CL)/TPL ratios decreased with cell growth (Fig. 2b,c,d,f). Although the ratios of all phospholipid classes markedly changed with cell growth, the cholesterol/TPL ratio and the TPL content were not significantly different among Day 3, Day 6 and Day 12 (Fig. 2h,i). In addition, the phospholipid compositions of the cells were compared at Day 3, Day 6 and Day 12. The ratios of the phospholipid classes were in the order PC > PE > PA > PS > SM ≥ PI > PG + CL at Day 3 in the early logarithmic phase of cell growth, whereas the ratios of PI and SM became higher than those of PS and PA at Day 12 in the stationary phase (Fig. 2j).

In addition, we examined the cell growth-associated changes in phospholipid class and cholesterol composition in human PLC/PRF/5 hepatoma cells, which have been established from the liver of a patient with primary liver cell carcinoma and produce hepatitis B surface antigen^45^. Similar to HepG2 cells, PLC/PRF/5 cells increased the PC/TPL ratio, reduced the PS/TPL, PA/TPL, and (PG + CL)/TPL ratios, and did not alter the cholesterol/TPL ratio during cell growth (Supplementary Fig. S2a,c,d,f,h,j). Unlike HepG2 cells, the TPL content decreased, the PE/TPL ratio increased, and the PI/TPL and SM/TPL ratios were not altered in PLC/PRF/5 cells during cell growth (Supplementary Fig. S2b,e,g,i). The phospholipid compositional changes during cell growth may partially differ among cell lines.

These observations suggested that the metabolism of all membrane phospholipid classes is dynamically controlled during cell growth, which may affect the proliferation, migration, morphology and functions of the cells. PA acts as a regulator of the mammalian target of rapamycin (mTOR), and the mTOR signaling pathway is involved in the control of cell growth through protein synthesis regulation^18,19,29^. The Ras-specific guanine-nucleotide exchange factor, son of sevenless (Sos), and the serine/threonine protein kinase, Raf-1, play crucial roles in cell growth. PA interacts with the Sos pleckstrin homology domain with high affinity and specificity, which is required for Ras activation^46^. Activated Ras recruits Raf-1 to the plasma membrane. Raf-1 selectively binds to PS and PA, which is important for the translocation and activation of Raf-1^47^. Activated Raf-1 sequentially phosphorylates and activates MEK and ERK kinases. On the other hand, the SH2 domain-containing phosphatase, SHP-1, suppresses cancer cell growth, and its activity is effectively stimulated by PA and PS^48^. At the final stage of cytokinesis, the surface exposure of PE on the cleavage furrow membrane may be important for the completion of cytokinesis^49^. PI is further phosphorylated to phosphoinositides, which regulate cell growth, cell survival, protein synthesis and vesicular trafficking as intracellular signaling mediators^21^. The cone-shaped phospholipids, PE, PA and CL, reduce the activation energy for negative curvature at membrane curves^38,50,51^, which may further lead to morphological changes of the cells and organelles. Together with cholesterol, SM induces the formation of lipid raft membrane microdomains, where acyl chains are well ordered and tightly packed, and diverse cellular processes are activated^52^.

### Alteration of gene expression for phospholipid biosynthesis in HepG2 cells during cell growth

**Figure 3:**
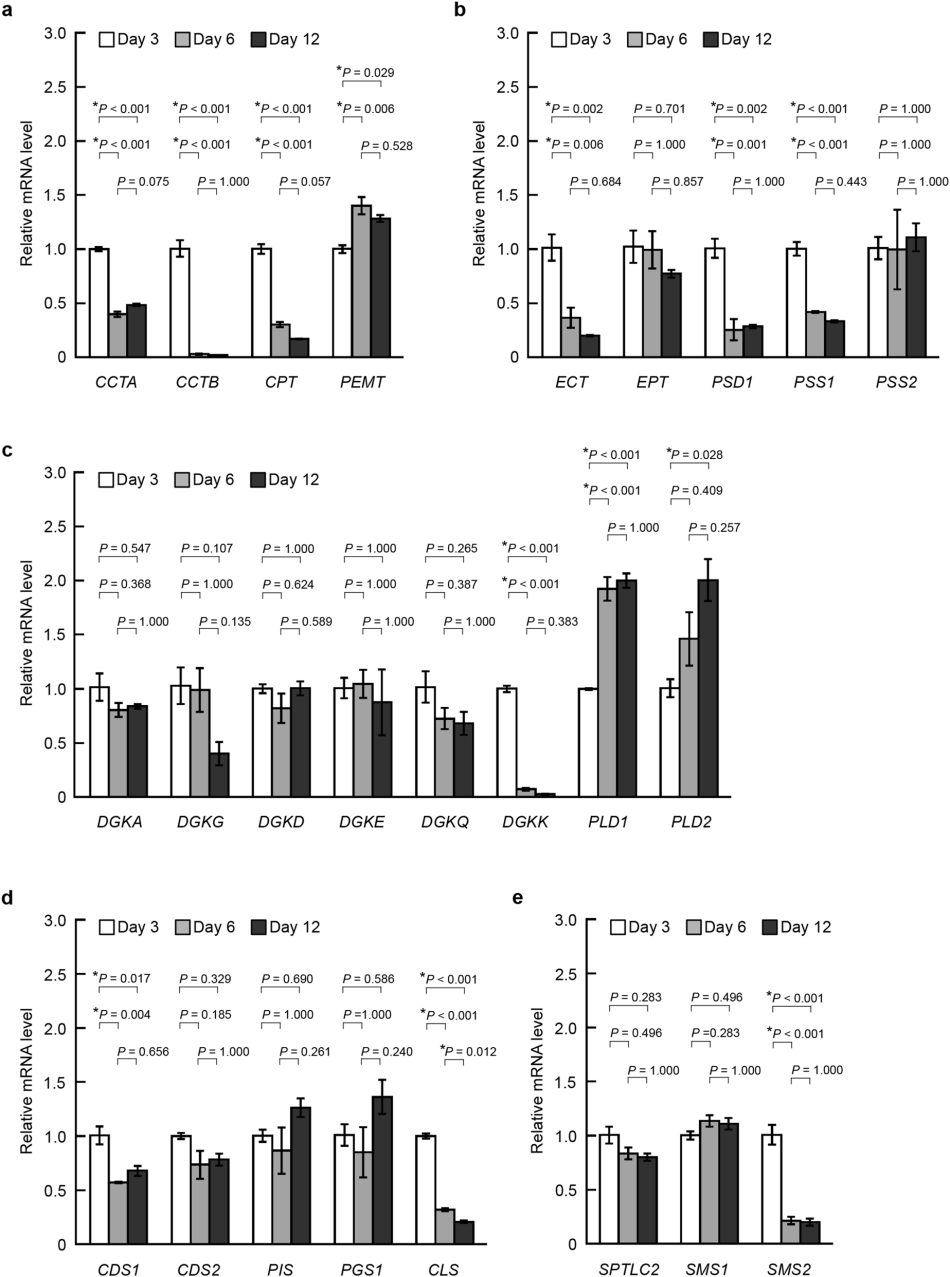
mRNA expression changes of phospholipid synthesis-related enzymes in HepG2 cells during cell growth. Relative mRNA expression of *CCTA*, *CCTB, CPT* and *PEMT* (**a**)*,* that of *ECT, EPT*, *PSD1*, *PSS1* and *PSS2* (**b**), that of *DGKA*, *DGKG, DGKD, DGKE, DGKQ, DGKK, PLD1* and *PLD2* (**c**), that of *CDS1*, *CDS2*, *PIS*, *PGS1* and *CLS* (**d**), and that of *SPTLC2*, *SMS1* and *SMS2* (**e**) in HepG2 cells at Day 3 (early logarithmic phase), Day 6 (late logarithmic phase) and Day 12 (stationary phase). The *Ct* value was normalized to the mean *Ct* value of *HPRT1* and *RPLP0*. The mRNA expression relative to the mRNA expression level at Day 3 was measured (mean ± S.E., n = 3, *P < 0.05, significantly different among Day 3, Day 6 and Day 12, one-way ANOVA followed by the Bonferroni test).

In mammalian cells, many enzymes participate in the biosynthesis of phospholipid classes, and phospholipid metabolic dysfunction is involved in carcinogenesis and tumor progression^18,21,29,53^. Next, to investigate how phospholipid class biosynthesis changes during cell growth, we evaluated the mRNA expression levels of phospholipid synthesis-related enzymes in HepG2 cells at Day 3, Day 6 and Day 12 by quantitative real-time PCR (qPCR) analyses (Fig. 3 and Supplementary Table S2). Among 32 candidate genes, *HPRT1* and *RPLP0* were selected as multiple reference genes (Supplementary Fig. S3). The mRNA expression levels of many enzymes at Day 6 and Day 12 changed compared with those at Day 3 (Fig. 3). As shown in Fig. 3a, the expression levels of genes for PC synthesis-related enzymes, *CCTA*, *CCTB* and *CPT*, in the cells at Day 6 and Day 12 significantly decreased compared with those at Day 3, whereas *PEMT* expression levels at Day 6 and Day 12 were slightly higher than that at Day 3. We also assessed the relative mRNA expression levels of PE and PS biosynthesis-related enzymes (Fig. 3b). The expression levels of *ECT*, *PSD1* and *PSS1* at Day 6 and Day 12 were lower than those at Day 3, and there were no differences in the expression levels of *EPT* or *PSS2* among Day 3, Day 6 and Day 12. As shown in Fig. 3c, among genes for six isoforms of DGKs (*DGKA*, *DGKG*, *DGLD*, *DGKE, DGKQ and DGKK*), only *DGKK* exhibited a significant decrease in the expression level at Day 6 and Day 12, although the expression of *DGKB*, *DGKZ*, *DGKH* and *DGKI* was not detected by the qPCR analysis. In contrast, the relative expression levels of *PLD1* and *PLD2* increased with cell growth. Moreover, the expression levels of *CDS1* and *CLS* decreased at Day 6 and Day 12, whereas there were no significant changes in those of *CDS2*, *PIS* and *PGS1* (Fig. 3d). Among the genes for SM synthesis, HepG2 cells exhibited decreased expression levels of *SMS2*, but not *SPTLC2* and *SMS1*, at Day 6 and Day 12 (Fig. 3e).

These results suggested that the marked changes in phospholipid class composition in HepG2 cells from the early logarithmic phase to stationary phase of cell growth were partly, but not entirely, due to the alteration in expression of phospholipid synthesis-related enzymes. The decrease in the PE ratio during cell growth (Fig. 2b) may be caused by the downregulation of *ECT* and *PSD1* (Fig. 3b). The cell growth-dependent reduction of the PS ratio (Fig. 2c) was likely caused by *PSS1* downregulation (Fig. 3b).

### Alteration in phospholipid compositions of organellar membranes from HepG2 cells during cell growth

**Figure 4:**
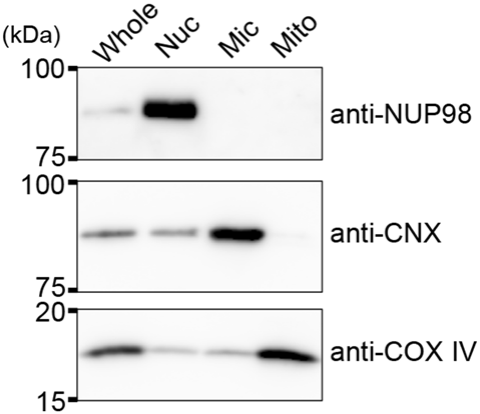
Isolation of organellar fractions from HepG2 cells. The whole-cell (Whole), purified nuclear (Nuc), microsomal (Mic) and mitochondrial (Mit) fractions (2.0 μg of protein) were separated by 7% or 15% SDS-PAGE and then immunoblotted with anti-NUP98, anti-CNX or anti-COX IV antibody. NUP98, CNX and COX IV are markers of the nucleus, ER and mitochondria, respectively. Three blots were cropped from different PVDF membranes. The full-length blots are presented in Supplementary Fig. S4.

To further understand the relationships between cell growth and phospholipid metabolism in HepG2 cells, we investigated the alteration of phospholipid class compositions in intracellular organelles during cell growth. We isolated nuclear, microsomal and mitochondrial membrane fractions by differential ultracentrifugation, and confirmed the purities of these fractions by immunoblotting for nuclear pore complex protein 98 (NUP98), calnexin (CNX) and cytochrome *c* oxidase subunit IV (COX IV), which are markers of the nuclear envelopes, ER and mitochondria, respectively (Fig. 4 and Supplementary Fig. S4).

**Figure 5:**
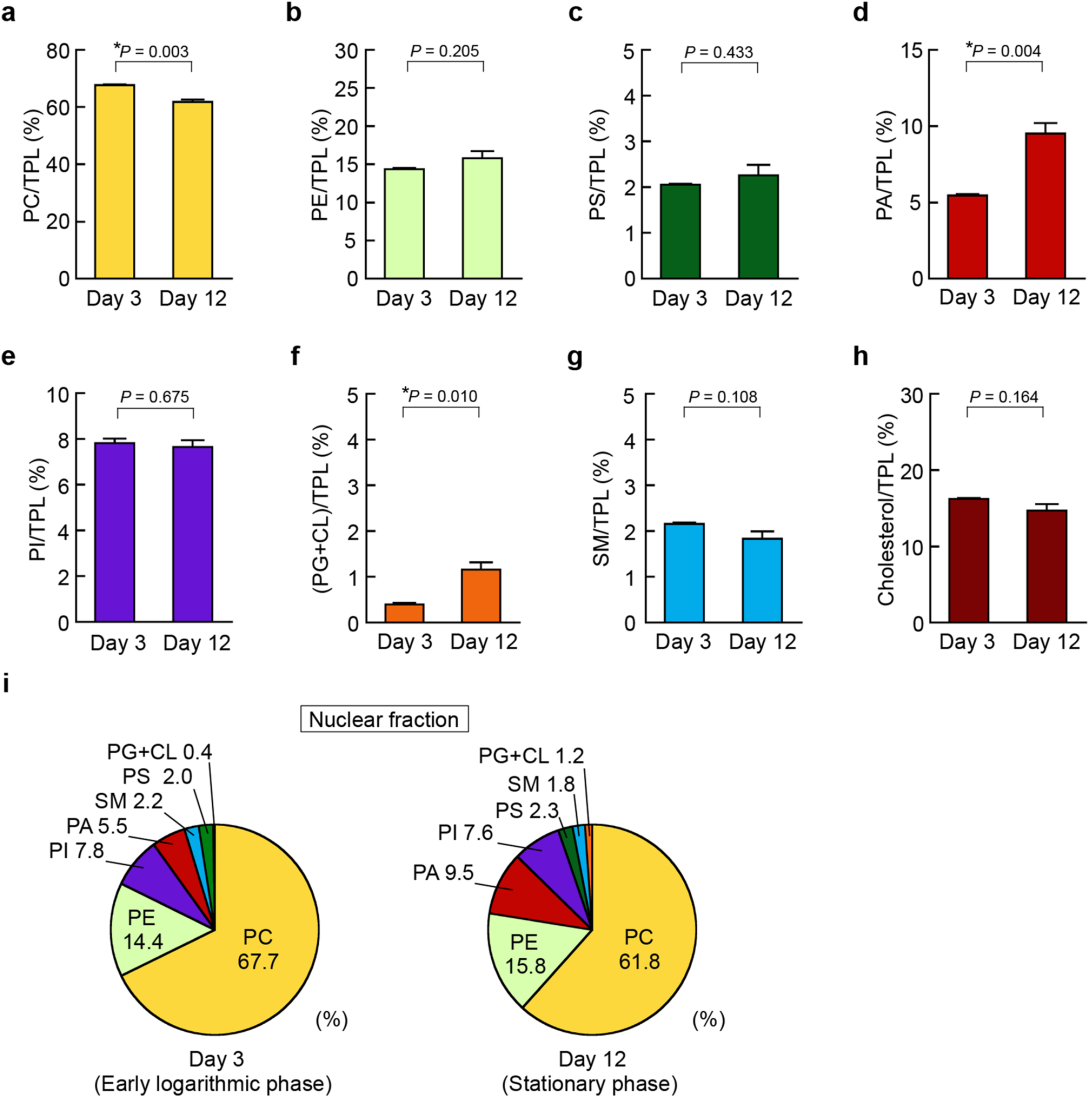
Alteration of nuclear phospholipid composition in HepG2 cells during cell growth. HepG2 cells were seeded at a density of 5.0 × 10^4^ cells/cm^2^ in 150-cm^2^ flasks and cultured in DMEM containing 10% FBS at 37 °C for the indicated days. At Day 3 (early logarithmic phase) and Day 12 (stationary phase), nuclear fractions were isolated from the cells, and nuclear lipids were extracted. The total phospholipid (TPL) content was calculated as the sum of PC, PE, PS, PA, PI, PG + CL and SM contents. The ratios of PC/TPL (**a**), PE/TPL (**b**), PS/TPL (**c**), PA/TPL (**d**), PI/TPL (**e**), (PG + CL)/TPL (**f**), SM/TPL (**g**) and cholesterol/TPL (**h**) in the nuclear fractions purified from the cells at Day 3 and Day 12 were determined by the enzymatic measurements (mean ± S.E., n = 3, *P < 0.05, significantly different between Day 3 and Day 12, unpaired two-tailed Student’s *t*-test). (**i**) The phospholipid compositions of the purified nuclear fractions from HepG2 cells at Day 3 and Day 12 are shown as pie charts.

**Figure 6:**
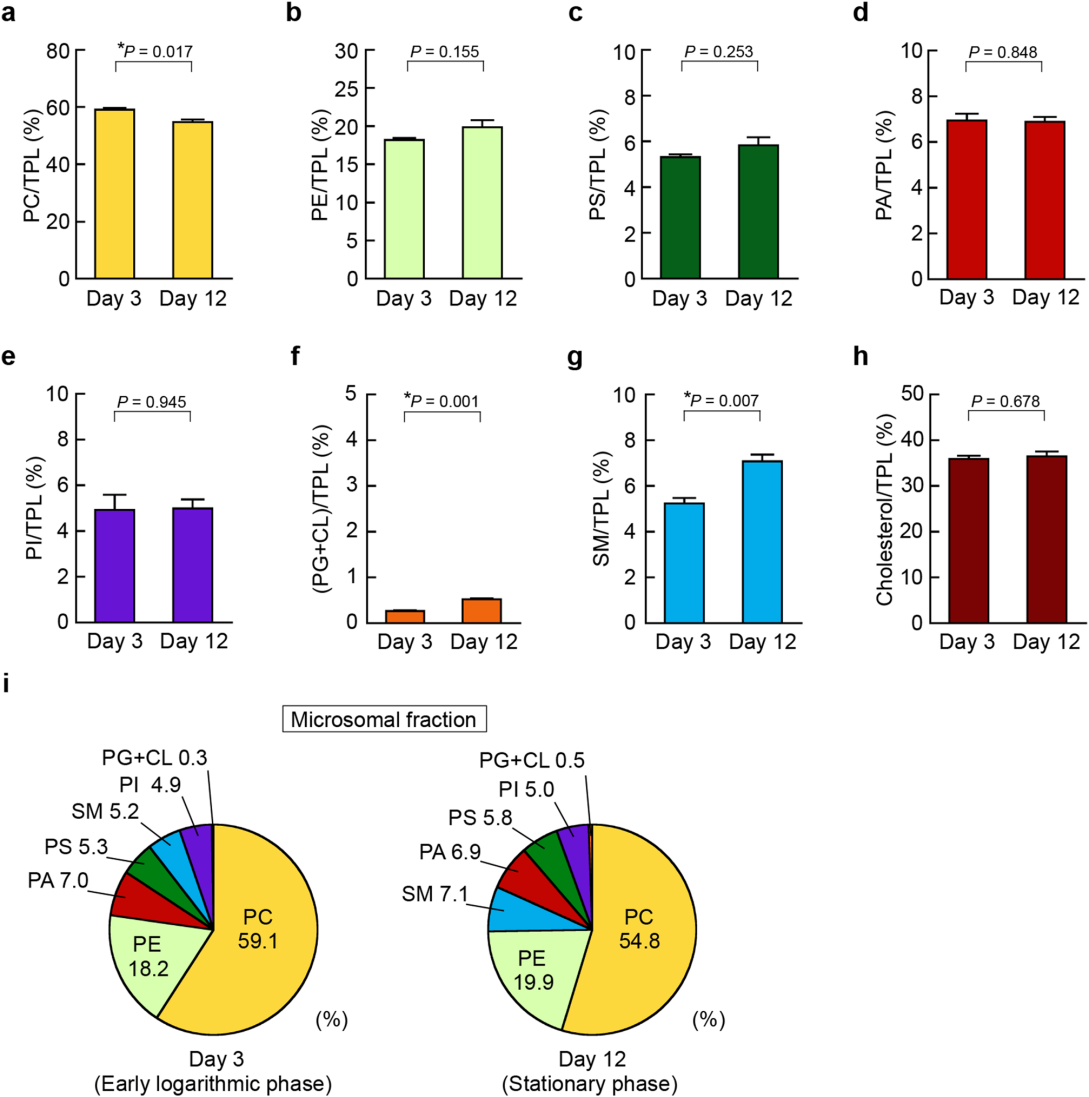
Alteration of microsomal phospholipid composition in HepG2 cells during cell growth. HepG2 cells were seeded at a density of 5.0 × 10^4^ cells/cm^2^ in 150-cm^2^ flasks and cultured in DMEM containing 10% FBS at 37 °C for the indicated days. At Day 3 (early logarithmic phase) and Day 12 (stationary phase), microsomal fractions were isolated from the cells, and microsomal lipids were extracted. The total phospholipid (TPL) content was calculated as the sum of PC, PE, PS, PA, PI, PG + CL, and SM contents. The ratios of PC/TPL (**a**), PE/TPL (**b**), PS/TPL (**c**), PA/TPL (**d**), PI/TPL (**e**), (PG + CL)/TPL (**f**), SM/TPL (**g**) and cholesterol/TPL (**h**) in the microsomal fractions purified from the cells at Day 3 and Day 12 were determined by the enzymatic measurements (mean ± S.E., n = 3, *P < 0.05, significantly different between Day 3 and Day 12, unpaired two-tailed Student’s *t*-test). (**i**) The phospholipid compositions of the purified microsomal fractions from HepG2 cells at Day 3 and Day 12 are shown as pie charts.

**Figure 7:**
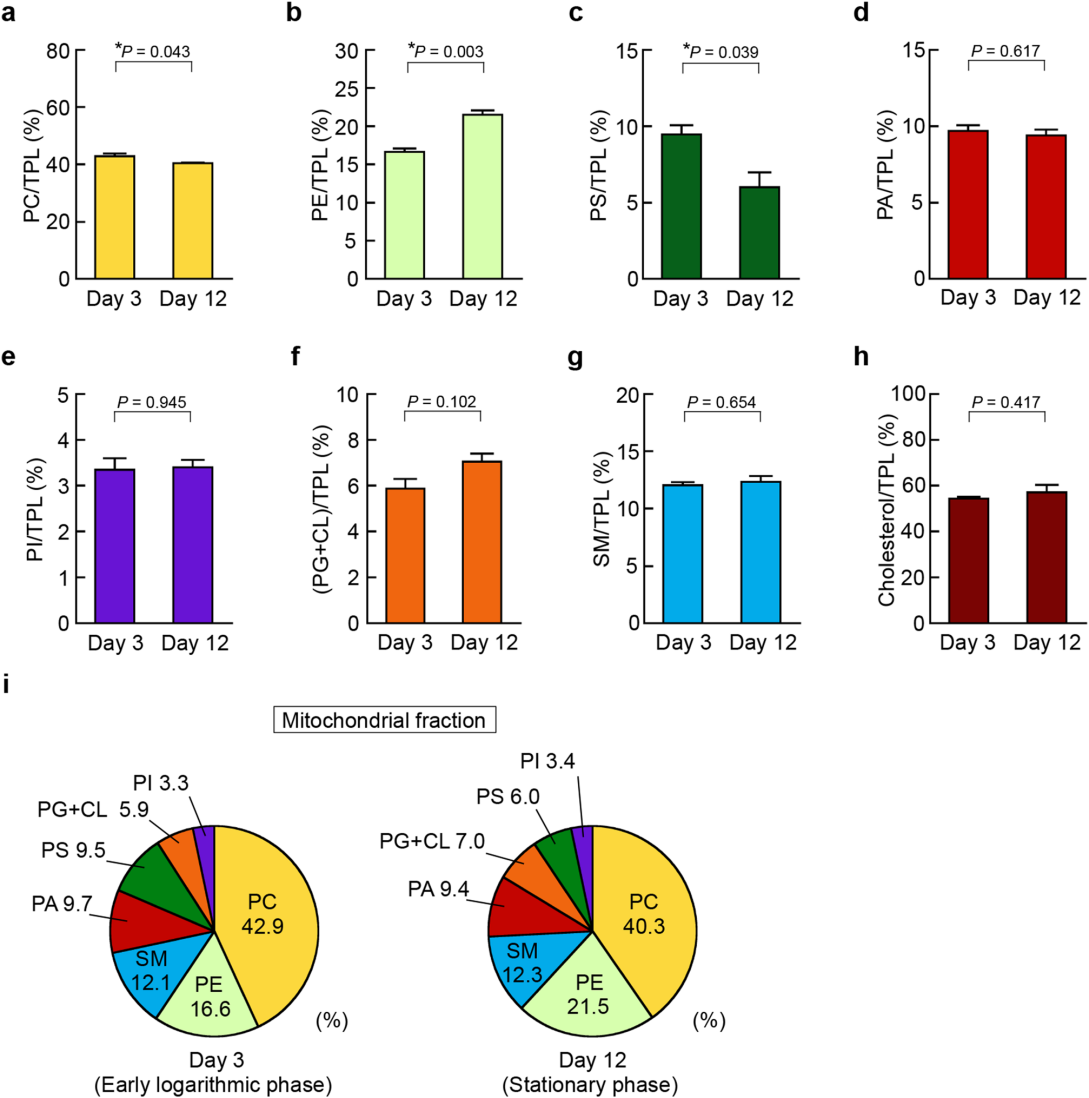
Alteration of mitochondrial phospholipid composition in HepG2 cells during cell growth. HepG2 cells were seeded at a cell density of 5.0 × 10^4^ cells/cm^2^ in 150-cm^2^ flasks and cultured in DMEM containing 10% FBS at 37 °C for the indicated days. At Day 3 (early logarithmic phase) and Day 12 (stationary phase), mitochondrial fractions were isolated from the cells and mitochondrial lipids were extracted. The total phospholipid (TPL) content was calculated as the sum of PC, PE, PS, PA, PI, PG + CL, and SM contents. The ratios of PC/TPL (**a**), PE/TPL (**b**), PS/TPL (**c**), PA/TPL (**d**), PI/TPL (**e**), (PG + CL)/TPL (**f**), SM/TPL (**g**) and cholesterol/TPL (**h**) in the mitochondrial fractions purified from the cells at Day 3 and Day 12 were determined by the enzymatic measurements (mean ± S.E., n = 3, *P < 0.05, significantly different between Day 3 and Day 12, unpaired two-tailed Student’s *t*-test). (**i**) The phospholipid compositions of the purified microsomal fractions from HepG2 cells at Day 3 and Day 12 are shown as pie charts.

We evaluated the phospholipid class and cholesterol compositions in the purified organellar fractions at Day 3 in the early logarithmic phase and at Day 12 in the stationary phase of cell growth using enzymatic fluorometric assays (Figs. 5, 6, 7). In the nuclear fractions, the ratio of PC/TPL at Day 12 was decreased compared with that at Day 3 (Fig. 5a). Of note, the PA/TPL ratio was 1.74-fold higher in the nuclear fraction at Day 12 than that at Day 3 (Fig. 5d). The ratio of PG + CL also slightly increased with cell growth, however, they were very minor components in the nuclear fraction even at Day 12 (Fig. 5f). The PE/TPL, PS/TPL, PI/TPL, SM/TPL and cholesterol/TPL ratios in the nuclear fractions did not change during cell growth (Fig. 5b,c,e,g,h). In the nuclear fraction from the cells at Day 3 in the early logarithmic phase, the most abundant phospholipid was PC, followed in descending order by PE, PI, PA, SM, PS and PG + CL, whereas PA became the third most abundant phospholipid component in the nuclear fraction at Day 12 in the stationary phase (Fig. 5i).

The microsomal fraction includes vesicles derived from the ER and Golgi apparatus, which are the predominant sites of phospholipid and cholesterol biosyntheses^26,36,54^. In the microsomal fractions, the ratios of PE, PS, PA, PI and cholesterol to TPL did not significantly change from Day 3 in the early logarithmic phase to Day 12 in the stationary phase (Fig. 6b–e,h). The microsomal fraction at Day 12 had a slightly lower PC/TPL ratio than that at Day 3 (Fig. 6a). Similar to the nuclear membranes, the increase in the PG + CL ratio during cell growth was significant in the microsomal membranes, but the ratio remained quite low even at Day 12 (Fig. 6f). Interestingly, the SM/TPL ratio in the microsomal fraction was 1.35-fold higher at Day 12 than at Day 3 (Fig. 6g). In the microsomal fraction from the cells at Day 3 in the early logarithmic phase, the phospholipid class ratios, from highest to lowest, were PC > PE > PA > PS ≥ SM ≥ PI > PG + CL, whereas the ratio of SM became higher than that of PS and similar to that of PA at Day 12 in the stationary phase (Fig. 6i).

In the mitochondrial fraction during cell growth, the PE/TPL ratio increased, but the PS/TPL ratio decreased (Fig. 7b,c). On the other hand, the PC/TPL ratio only slightly decreased, and the ratios of PA, PI, SM and cholesterol to TPL did not significantly change from Day 3 in the early logarithmic phase to Day 12 in the stationary phase (Fig. 7a,d,e,g,h). In contrast to the nuclear and microsomal fractions, there was no significant difference in the mitochondrial (PG + CL)/TPL ratios between Day 3 and Day 12, but the mitochondrial fractions contained high ratios of PG + CL (Fig. 7f). In the mitochondrial fractions, the most abundant phospholipid was PC, followed in descending order by PE, SM, PA, PS, PG + CL and PI at Day 3 in the early logarithmic phase, whereas the ratio of PS became lower at Day 12 in the stationary phase (Fig. 7i).

Although phospholipids are synthesized mainly in the ER and mitochondria in mammalian cells, PC and PA are also produced in the nuclear envelopes^18,26,29^. CCTs are rate-limiting enzymes in the CDP-choline pathway for PC biosynthesis^26^. The cell growth-related decrease in the PC ratio in the nuclear envelops may be explained by the down-regulation of *CCTA* (Figs. 3a, 5a) because CCTα, but not CCTβ, is localized primarily in the nucleus^26^. CPT is an integral ER membrane protein^26^. Thus, the reduction in the microsomal PC ratio in the stationary phase may be caused by lower expression levels of *CCTB* and/or *CPT*. In contrast to the nuclear, microsomal and mitochondrial fractions, the PC ratio in the whole cells slightly but significantly increased during cell growth (Fig. 2a), which may be caused by the increase in the PC ratio in other cellular compartments such as the plasma membrane.

The growth-arrested cells exhibit a higher coupling state of oxidative phosphorylation than the proliferating cells^55^. During cell growth to confluence, there is an increase in the proportion of mitochondria to be shorter bead-like forms and more randomly aligned^56^. Mitochondrial cristae are tube-like extensions of the mitochondrial inner membrane, which is highly enriched in negative curvature phospholipids, PE and CL^57^. Mitochondrial PE is essential for the correct morphology of mitochondria^58,59^. PE is produced from PS by PSD in mitochondrial inner membranes^27,35^. As shown in Figs. 3b, 7b,c, the mitochondrial PE/TPL ratio increased and the mitochondrial PS/TPL ratio decreased with cell growth despite *PSD1* downregulation, raising the possibility that the export of mitochondrial PE to other organellar membranes and the import of PS to mitochondrial membranes were suppressed in the stationary phase. The counter-transport of PS and PI(4)P between the ER and plasma membranes is mediated by oxysterol-binding protein-related proteins 5 and 8, which have also been reported to be localized to the ER-mitochondria contact sites^60,61^. However, the mechanism underlying the translocation of PE and PS between mitochondria and other organelles is largely unclear.

The production of PA from PC is catalyzed by the membrane-bound PLDs. PLD1 is localized to the perinuclear region, Golgi, ER and late endosomes, whereas PLD2 is localized to the plasma membrane^29,50^. The increase in the ratio of PA in nuclear membranes during cell growth (Fig. 5d) may be due to the upregulation of *PLD1*. DGK isoforms also function in the production of PA around nuclear envelopes. Most DGK isoforms are cytosolic in mammalian cells, but the translocations of DGKα, DGKγ, DGKθ, DGKζ and DGKι from the cytosol to the nucleus regulate their spatiotemporal activation^18^. In HepG2 cells, the mRNA expression levels of *DGKA*, *DGKG* and *DGKQ* were not regulated by cell growth (Fig. 3c), and those of *DGKZ* and *DGKI* were not detectable. Thus, DGKs were likely not responsible for the increase in the PA ratio in nuclear envelopes in the stationary phase of cell growth.

Mitochondria are exclusive sites for the biosynthesis of PG and CL, which are required for mitochondrial energy production^20,33,35^. Cell growth had no significant effect on the mitochondrial ratio of PG + CL despite the downregulation of *CLS* (Figs. 3d, 7f). Therefore, the ratio of PG + CL in mitochondrial membranes was suggested to be strictly maintained during the cell growth, which may be crucial for the correct mitochondrial functions.

The increase in the microsomal SM ratio during cell growth is shown in Fig. 6g. On the other hand, *SMS2* was down-regulated, and *SPTLC2* and *SMS1* were constantly expressed during cell growth (Fig. 3e). SM is formed from ceramide and the phosphocholine group of PC by SMS1 at the Golgi membranes or by SMS2 at the plasma membrane^36^. However, the regulatory mechanism to translocate SM into the ER remains unclear.

Sterol regulatory element-binding proteins (SREBPs) form complexes with SREBP-cleavage activating protein (SCAP) at the ER membranes and control the membrane cholesterol homeostasis^62^. When the cholesterol concentration increases in the ER membranes, the binding of cholesterol to the sterol-sensing domain of SCAP induces the association of SCAP with Insig, which prevents the transport of SREBP/SCAP complexes to the Golgi and the proteolytic activation of SREBP^62^. In HepG2 cells, the ratios of cholesterol to TPL were different among the nuclear, microsomal and mitochondrial membranes (~ 15%, ~ 36% and ~ 55%, respectively), but the cholesterol ratio in each membrane fraction was not affected by the cell growth (Figs. 5, 6, 7). It is possible that machineries to tightly regulate the membrane cholesterol ratio exist in the nuclear envelopes and mitochondria in addition to the ER.

We have previously investigated the relationships between expression levels of phospholipid synthesis-related enzymes and phospholipid class composition using stably transfected HEK293 cells. The increase in PEMT expression leads to the increased PC content and the decreased PE content^40^. The cellular PS content increases due to the overexpression of PSS1^41^. The increased expression of PGS1 results in the elevated mitochondrial content of PG + CL^43^. The increase in the expression of PIS, CDS1 or CDS2 induces an increase in the PI ratio in microsomes, but not in mitochondria^44^. Thus, during cell growth, the altered expression levels of phospholipid synthesis-related enzymes may affect the cellular and/or organellar phospholipid class composition in HepG2 cells.

In conclusion, by using enzymatic fluorometric methods for quantifying phospholipid classes, we demonstrated the changes in phospholipid compositions in HepG2 cells and their intracellular organelles during cell growth using enzymatic fluorometric methods to quantify phospholipid classes. All phospholipid class ratios, but not the cholesterol ratio, markedly changed depending on cell growth. The higher PA ratio and lower PC ratio in the nuclear membrane, the higher SM ratio in the microsomal membrane and the higher PE ratio and lower PS ratio in the mitochondrial membrane were characteristic of the stationary phase compared with the early logarithmic phase. In addition, qPCR analysis revealed that the mRNA expression levels of many phospholipid synthesis-related enzymes, particularly *CCTA*, *CCTB*, *CPT*, *ECT*, *PSD1*, *PSS1*, *DGKK*, *PLD1*, *PLD2*, *CLS* and *SMS2*, were affected by cell growth. This study provides fundamental and valuable information to further understand the mechanisms underlying cancer cell growth and lipid metabolism.

## Methods

### Cell culture

HepG2 cells were purchased from Riken BioResource Center (Tsukuba, Japan). HepG2 cells were cultured in DMEM containing 10% heat-inactivated fetal bovine serum (FBS) in 5% CO_2_ at 37 °C, and confluent cells were subcultured at a split ratio of 1:5 after trypsin–EDTA treatment (Nacalai Tesque, Kyoto, Japan). For experiments, HepG2 cells were seeded at a density of 5.0 × 10^4^ cells/cm^2^ in six-well plates (4.8 × 10^5^ cells/well) (Thermo Fisher Scientific, Rockford, IL, USA), in 75-cm^2^ flasks (3.75 × 10^6^ cells/flask) (TPP Techno Plastic Products, Trasadingen, Switzerland) or in 150-cm^2^ flasks (7.50 × 10^6^ cells/flask) (TPP Techno Plastic Products), and incubated in DMEM with 10% FBS.

### Cell growth experiments

HepG2 cells were seeded at a density of 5.0 × 10^4^ cells/cm^2^ in six-well plates and cultured in DMEM with 10% FBS for up to 14 days. The culture medium was changed every other day. Cells were washed and scraped with cold phosphate-buffered saline (PBS). To prepare whole cell lysates, cells were sonicated using an Ultrasonic Disruptor UR-20P (Tomy Seiko, Tokyo, Japan) and lysed with 1% Triton X-100 in PBS. The concentrations of total DNA and protein in cell lysates were measured using a Quant-iT dsDNA BR Assay Kit (Invitrogen, Carlsbad, CA, USA) and a BCA protein assay kit (Thermo Fisher Scientific, Rockford, IL, USA), respectively.

### RNA isolation and cDNA synthesis

HepG2 cells were seeded at a density of 5.0 × 10^4^ cells/cm^2^ in six-well plates and cultured in DMEM with 10% FBS for 3, 6 and 12 days. Cells were harvested and pelleted followed by total RNA isolation using an SV Total RNA Isolation System (Promega, Madison, WI). The concentration of total RNA was measured using a Quant-iT RNA BR Assay Kit (Invitrogen, Carlsbad, CA, USA). To obtain cDNA, 0.5 μg of total RNA was used as the template for reverse transcription using ReverTra Ace qPCR RT Master Mix with gDNA Remover (Toyobo, Osaka, Japan) according to the manufacturer’s instructions. cDNA was diluted 1:20 in nuclease-free water before being used as a template for qPCR.

### qPCR

TaqMan Custom Array Fast Plate (Applied Biosystems, Foster City, CA, USA) was used to evaluate the expression levels of phospholipid synthesis-related enzymes in HepG2 cells. TaqMan Gene Expression assays in the plate are shown in Supplementary Table S2. Among 32 candidate genes, *HPRT1* and *RPLP0* were selected as reference genes using the TaqMan Array Human Endogenous Control Plate, Fast 96-well (Applied Biosystems) by NormFinder and BestKeeper algorithms^63,64^ (Supplementary Fig. S2). qPCR was carried out using the StepOnePlus Real-Time PCR System and StepOne Software v2.3 (Applied Biosystems). PCR reactions were performed with cDNA as the template and with TaqMan Fast Advanced Master Mix (Applied Biosystems). The cycler program was (i) 2 min at 50 °C, (ii) 20 s at 95 °C, and (iii) 40 cycles of 1 s at 95 °C and 20 s at 60 °C. The relative mRNA expression was calculated by the comparative Ct (ΔΔCt) method^65^. The values of the threshold cycle (*Ct*) were obtained from three separate experiments (n = 3), and normalized to the mean of *Ct* values of *HPRT1* and *RPLP0*. The mRNA expression relative to that at Day 3 (2^−ΔΔCt^) was calculated.

### Measurement of phospholipid and cholesterol contents

To measure the contents of cellular phospholipids and cholesterol, HepG2 cells were seeded at a density of 5.0 × 10^4^ cells/cm^2^ in 75-cm^2^ flasks and cultured in DMEM with 10% FBS for 3, 6 and 12 days. Cells were washed, scraped with cold PBS and sonicated to prepare whole-cell homogenates. To measure the contents of phospholipids and cholesterol in purified nuclear, mitochondrial and microsomal fractions, cells were seeded at a density of 5.0 × 10^4^ cells/cm^2^ in 150-cm^2^ flasks and cultured in DMEM with 10% FBS for 3 and 12 days. Cells were washed with cold PBS and homogenized in the homogenization buffer (225 mM mannitol, 75 mM sucrose, 30 mM Tris–HCl and 100 μM EGTA, pH 7.4) using a Potter-Elehjem homogenizer, and the homogenate was centrifuged for 5 min at 600×*g* to obtain the pellet containing nuclei and the supernatant containing mitochondria and microsomes. Purified nuclear, mitochondrial and microsomal fractions were isolated from six flasks of cells at Day 3 and from one flask of cells at Day 12 according to the method previously described^66,67^. Purified organellar fractions were washed once and suspended in 5 mM HEPES buffer (pH 7.4).

Cellular and organellar lipids were extracted by the method of Folch as described previously^44,68^. Lipid extract was dissolved by the addition of 1% Triton X-100. The contents of PC, PE, PS, PA, PI, PG + CL, SM and cholesterol in the extracts were quantified by enzymatic assays using combinations of specific enzymes and Amplex Red as previously described^13,38–41,43,44^. The total phospholipid (TPL) content was calculated as the sum of PC, PE, PS, PA, PI, PG + CL and SM contents.

We confirmed the recovery of phospholipid classes after the Folch extraction method (see Supplementary Methods). As depicted in Supplementary Table S3, PC, PE, PG, CL and SM were almost completely recovered by the Folch method. Although the majority (> 80%) of PS, PA and PI molecules were recovered after the Folch method, the cellular contents of PS, PA and PI may have been slightly underestimated. Lysophosphatidylcholine and sphingosylphosphocholine are not detected by the enzymatic measurements, but lysophosphatidylethanol (LPE), lysophosphatidylserine (LPS), lysophosphatidic acid (LPA), lysophosphatidylinositol (LPI) and lysophosphatidylglycerol (LPG) can be measured by the enzymatic fluorometric assay for PE, PS, PA, PI and PG + CL, respectively^24^. After the Folch method, LPS, LPA, LPI and LPG were almost fully removed from the extract, whereas LPE was substantially recovered (Supplementary Table S3). It has been reported that, using supercritical fluid chromatography with a charged aerosol detector, LPE, but not PE, is not identified in the HepG2 extracts^69^. Therefore, the amounts of lysophospholipid classes are probably small enough to be ignored in the cellular lipid extracts.

### Immunoblotting

Whole cell homogenates and organellar fractions were lysed with 5 mM HEPES buffer containing 1% Triton X-100 and protease inhibitors (100 μg/ml (*p*-amidinophenyl)methanesulfonyl fluoride, 10 μg/ml leupeptin and 2 μg/ml aprotinin). Immunoblotting was performed as previously described^44^. The blotted PVDF membranes were incubated with monoclonal anti-NUP98 antibody C39A3 (1:2000 dilution; Cell Signaling Technology, Danvers, MA, USA), monoclonal anti-COX IV antibody 3E11 (1:20,000; Cell Signaling Technology) or polyclonal anti-CNX antibody (1:10,000; Enzo Life Sciences, Farmingdale, NY, USA), followed by incubation with horseradish peroxidase (HRP)-conjugated goat anti-mouse IgG (1:5000; Invitrogen) or HRP-conjugated goat anti-rabbit IgG (1:10,000; Merck Millipore). The image was obtained using Amersham ECL Prime Western Blotting Detection Reagent and an ImageQuant LAS 4000 mini biomolecular imager (GE Healthcare, Buckinghamshire, UK).

### Statistical analysis

The results are given as the means ± S.E.s of three biologically independent experiments unless otherwise indicated. Statistical significance was assessed by the unpaired Student’s *t*-test or one-way ANOVA followed by the Bonferroni test. Differences were considered significant at *P* < 0.05 (two-tailed).

## Supplementary Information

Supplementary Information.
