# Supplementary material for: Alterations in cellular and organellar phospholipid compositions of HepG2 cells during cell growth
Source: Sci Rep. 2021 Feb 1;11:2731. doi: 10.1038/s41598-021-81733-3 (PMC7851136; doi:10.1038/s41598-021-81733-3)
Supplement: Supplementary file 1 — Supplementary Information. [file 41598_2021_81733_MOESM1_ESM.pdf]

Supplementary Information

Alterations in cellular and organellar phospholipid compositions of HepG2 cells during cell growth

Tokuji Tsuji[1], Shin-ya Morita[1,*], Yoshinobu Nakamura[1], Yoshito Ikeda[1], Taiho Kambe[2], Tomohiro Terada[1]

[1]Department of Pharmacy, Shiga University of Medical Science Hospital, Otsu City, Shiga 520-2192, Japan. [2]Graduate School of Biostudies, Kyoto University, Kyoto 606-8502, Japan.

*Correspondence and requests for materials should be addressed to S.-y.M. (email: smorita@belle.shiga-med.ac.jp)

Supplementary Methods

Measurement of phospholipid and cholesterol contents in PLC/PRF/5 cells.

PLC/PRF/5 cells were obtained from the Japanese Collection of Research Bioresources Cell Bank (Tsukuba, Japan). PLC/PRF/5 cells were cultured in DMEM containing 10% heat-inactivated fetal bovine serum (FBS) in 5% CO_2 at 37°C. To measure the contents of cellular phospholipids and cholesterol, PLC/PRF/5 cells were seeded at a density of 2.5 × 10^3 cells/cm^2 in 10-cm dishes and cultured in DMEM with 10% FBS for 3 and 12 days. Cells were washed, scraped with cold PBS and sonicated to prepare whole-cell homogenates. Cellular and organellar lipids were extracted by the method of Folch as described previously[1, 2]. Lipid extract was dissolved by the addition of 1% Triton X-100. The contents of PC, PE, PS, PA, PI, PG + CL, SM and cholesterol in the extracts were quantified by enzymatic assays using combinations of specific enzymes and Amplex Red as previously described[3]. The total phospholipid (TPL) content was calculated as the sum of PC, PE, PS, PA, PI, PG + CL and SM contents.

Recovery of phospholipids after Folch extraction method.

PC from chicken egg yolk was obtained from Nacalai Tesque (Kyoto, Japan). PE from chicken egg yolk, PS from bovine brain, PA sodium salt from chicken egg yolk, CL sodium salt from bovine heart, SM from chicken egg yolk, LPE from chicken egg yolk, oleoyl LPA sodium salt, and LPI sodium salt from soybean were purchased from Sigma-Aldrich (St. Louis, MO, USA). PI from bovine milk was purchased from Nagara Science (Gifu, Japan). Oleoyl LPS sodium salt and oleoyl LPG sodium salt were obtained from Avanti Polar Lipids (Alabaster, AL, USA). PG from chicken egg yolk was purchased from NOF (Tokyo, Japan).

Phospholipids were extracted from the samples by the method of Folch[1, 2]. In brief, 4.0 ml of chloroform/methanol (2:1) solution was added to 1 ml of the sample solution and vortexed. After vortexing, the sample stood overnight at 4°C. The phase split was completed by centrifugation. The upper aqueous phase and the interfacial material were removed carefully. The recovered lower organic phase was washed with 1 ml of H_2O. The upper phase was removed again, and the organic solvent of the lower phase was evaporated. The evaporated sample was dissolved in 200 μl of 1%

Triton X-100. The contents of PC, PE, PS, PA, PI, PG, CL, SM, LPE, LPS, LPA, and LPG in the extracts were measured by enzymatic assays[3].

References

1. Folch, J., Lees, M. & Sloane Stanley, G.H. A simple method for the isolation and purification of total lipides from animal tissues. *J. Biol. Chem.* **226**, 497-509 (1957).
2. Tsuji, T., Morita, S.Y., Ikeda, Y. & Terada, T. Enzymatic fluorometric assays for quantifying all major phospholipid classes in cells and intracellular organelles. *Sci. Rep.* **9**, 8607 (2019).
3. Morita, S.Y., Tsuji, T. & Terada, T. Protocols for enzymatic fluorometric assays to quantify phospholipid classes. *Int. J. Mol. Sci.* **21**, 1032 (2020).

Supplementary Table S1. Phospholipid and cholesterol contents in HepG2 cells.

	Day 3	Day 6	Day 12
	(nmol/mg protein)	(nmol/mg protein)	(nmol/mg protein)
PC	113.37 ± 1.90	121.89 ± 1.35	120.55 ± 0.75
PE	39.28 ± 0.35	37.87 ± 0.50	37.94 ± 0.49
PS	13.32 ± 0.33	12.03 ± 0.18	11.63 ± 0.05
PA	15.42 ± 0.27	10.35 ± 0.18	9.60 ± 0.09
PI	11.08 ± 0.09	15.14 ± 0.26	14.06 ± 0.36
PG + CL	7.14 ± 0.16	5.06 ± 0.07	5.16 ± 0.13
SM	11.29 ± 0.23	12.45 ± 0.44	13.73 ± 0.58
TPL	210.90 ± 2.96	214.79 ± 2.26	212.67 ± 1.75
Cholesterol	64.00 ± 0.51	64.35 ± 1.56	66.75 ± 0.24

Values are mean ± S.E. (n = 3).

PC: phosphatidylcholine, PE: phosphatidylethanolamine, PS: phosphatidylserine, PA: phosphatidic acid, PI: phosphatidylinositol, PG: phosphatidylglycerol, CL: cardiolipin, SM: sphingomyelin, TPL: total phospholipid.

Supplementary Table S2. Genes analyzed by qPCR.

Gene symbol	Protein Name	TaqMan Assay ID
DGKA	Diacylglycerol kinase α	Hs00176278_m1
DGKB	Diacylglycerol kinase β	Hs00391660_m1
DGKG	Diacylglycerol kinase γ	Hs00176315_m1
DGKD	Diacylglycerol kinase δ	Hs01114125_m1
DGKE	Diacylglycerol kinase ε	Hs00177537_m1
DGKZ	Diacylglycerol kinase ζ	Hs01586726_m1
DGKH	Diacylglycerol kinase η	Hs00410739_m1
DGKQ	Diacylglycerol kinase θ	Hs01092337_m1
DGKI	Diacylglycerol kinase ι	Hs01546427_m1
DGKK	Diacylglycerol kinase κ	Hs01385647_m1
PLD1	Phospholipase D1	Hs01111342_m1
PLD2	Phospholipase D2	Hs01093216_m1
CCTA (*PCYT1A*)	CTP:phosphocholine cytidylyltransferase α	Hs00192339_m1
CCTB (*PCYT1B*)	CTP:phosphocholine cytidylyltransferase β	Hs00191464_m1
CPT (*CHPT1*)	CDP-choline:diacylglycerol cholinephosphotransferase	Hs01012468_m1
PEMT	Phosphatidylethanolamine *N*-methyltransferase	Hs01002999_m1
ECT (*PCYT2*)	CTP:phosphoethanolamine cytidylyltransferase	Hs00161098_m1
EPT	CDP-ethanolamine:diacylglycerol ethanolaminephosphotransferase	Hs00378840_m1
PSD (*PISD*)	Phosphatidylserine decarboxylase	Hs00204966_m1
PSS1 (*PTDSS1*)	Phosphatidylserine synthase 1	Hs00207371_m1
PSS2 (*PTDSS2*)	Phosphatidylserine synthase 2	Hs00992453_m1
CDS1	CDP-diacylglycerol synthase 1	Hs00181633_m1
CDS2	CDP-diacylglycerol synthase 2	Hs00300881_m1
PIS (*CDIPT*)	Phosphatidylinositol synthase	Hs00197004_m1
PGS1	Phosphatidylglycerophosphate synthase 1	Hs00922697_m1
CLS (*CRLS1*)	Cardiolipin synthase	Hs00219512_m1
SMS1 (*SGMS1*)	Sphingomyelin synthase 1	Hs00983630_m1
SMS2 (*SGMS2*)	Sphingomyelin synthase 2	Hs00380453_m1
SPTLC2	Serine palmitoyltransferase long chain base subunit 2	Hs01027014_m1
HPRT1	Hypoxanthine phosphoribosyltransferase 1	Hs99999909_m1
RPLP0	Ribosomal protein lateral stalk subunit P0	Hs99999902_m1

Supplementary Table S3. Recovery of phospholipids after Folch extraction method.

Phospholipid class	Recovery (%)
PC	97.6 ± 1.7
PE	99.5 ± 3.0
PS	83.0 ± 1.7
PA	88.8 ± 1.4
PI	86.1 ± 2.6
PG	95.3 ± 1.2
CL	98.1 ± 1.3
SM	97.4 ± 2.7
LPE	86.0 ± 1.3
LPS	1.9 ± 0.5
LPA	2.7 ± 0.3
LPI	0.0 ± 0.0
LPG	2.8 ± 0.5

Values are mean ± S.D. (n = 4).

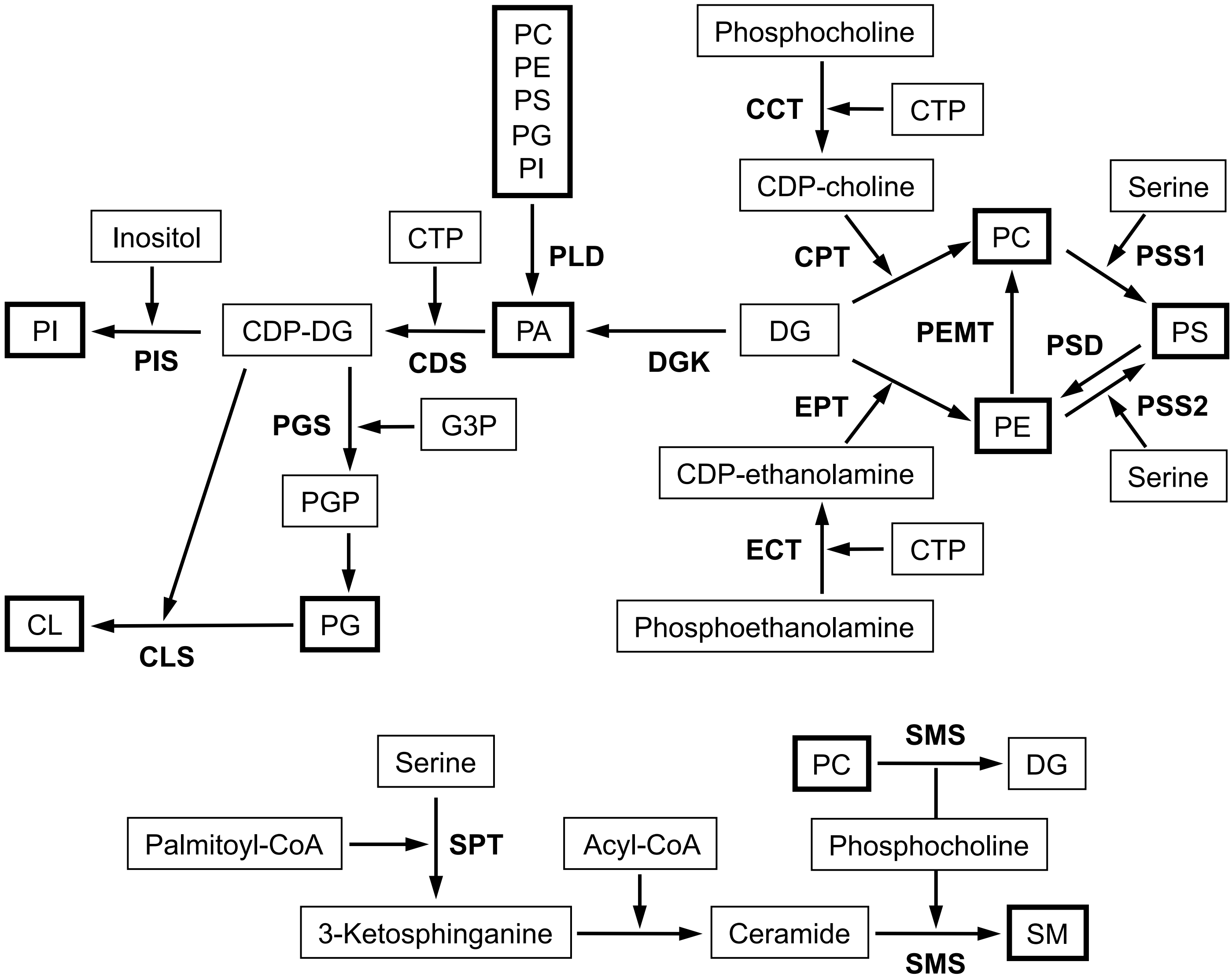

Supplementary Figure S1. Pathways for biosynthesis of major phospholipid classes in mammalian cells. CCT, CTP:phosphocholine cytidylyltransferase; CDS, CDP-diacylglycerol synthase; CL, cardiolipin; CLS, cardiolipin synthase; CPT, CDP-choline:diacylglycerol cholinephosphotransferase; DG, diacylglycerol; DGK, diacylglycerol kinase; ECT, CTP:phosphoethanolamine cytidylyltransferase; EPT, CDP-ethanolamine:diacylglycerol ethanolaminephosphotransferase; G3P, glycerol-3-phosphate; PA, phosphatidic acid; PC, phosphatidylcholine; PE, phosphatidylethanolamine; PEMT, phosphatidylethanolamine *N*-methyltransferase; PG, phosphatidylglycerol; PGP, phosphatidylglycerophosphate; PGS, phosphatidylglycerophosphate synthase; PI, phosphatidylinositol; PIS, phosphatidylinositol synthase; PLD, phospholipase D; PS, phosphatidylserine; PSD, phosphatidylserine decarboxylase; PSS, phosphatidylserine synthase; SM, sphingomyelin; SMS, sphingomyelin synthase; SPT, serine palmitoyltransferase.

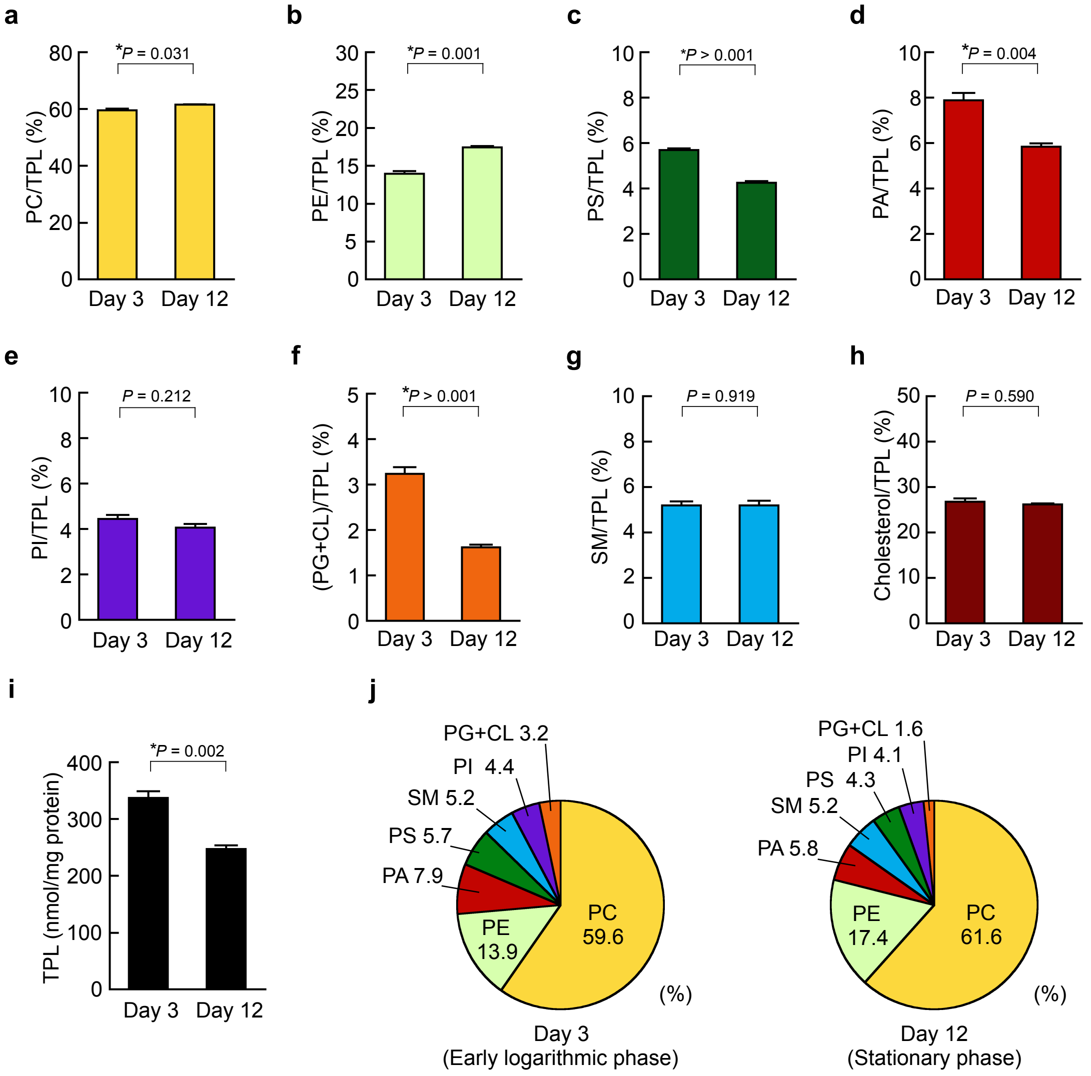

Supplementary Figure S2. Alteration of phospholipid composition in PLC/PRF/5 cells during cell growth. PLC/PRF/5 cells were seeded at a density of 2.5 × 10^3 cells/cm^2 in 10-cm dishes and cultured in DMEM containing 10% FBS at 37°C for the indicated days. At Day 3 (early logarithmic phase) and Day 12 (stationary phase), cellular lipids were extracted. The contents of PC, PE, PS, PA, PI, PG + CL, SM and cholesterol in PLC/PRF/5 cells were determined by the enzymatic measurements and protein assay. The ratios of PC/TPL (**a**), PE/TPL (**b**), PS/TPL (**c**), PA/TPL (**d**), PI/TPL (**e**), (PG + CL)/TPL (**f**), SM/TPL (**g**) and cholesterol/TPL (**h**) at Day 3 and Day 12 were evaluated. The TPL content (**i**) was calculated as the sum of PC, PC, PE, PS, PA, PI, PG + CL, and SM contents (**i**) (mean ± S.E., n = 3, *$P < 0.05$, significantly different between Day 3 and Day 12, unpaired two-tailed Student's *t*-test). (**j**) The phospholipid compositions in PLC/PRF/5 cells at Day 3 and Day 12 are shown as pie charts.

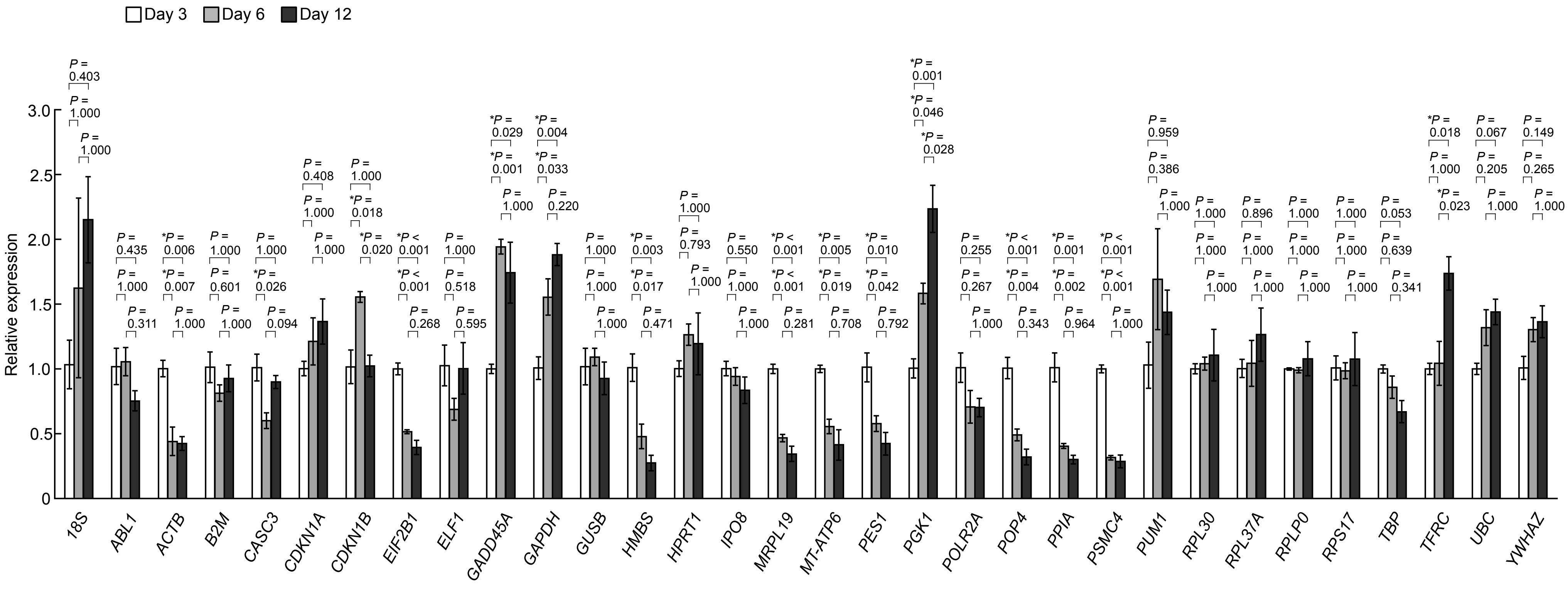

Supplementary Figure S3. Expression changes of 32 candidate reference genes in HepG2 cells during cell growth. The expression level of each gene at Day 3 (early logarithmic phase), Day 6 (late logarithmic phase) and Day 12 (stationary phase) was evaluated by *Ct* values. The expression level relative to that at Day 3 ($2^{-\Delta Ct}$) was calculated (mean ± S.E., n = 3, *$P < 0.05$, significantly different among Day 3, Day 6 and Day 12, one-way ANOVA followed by the Bonferroni test). The relative expression of 17 genes (*18S*, *ABL1*, *B2M*, *CDKN1A*, *ELF1*, *GUSB*, *HPRT1*, *IPO8*, *POLR2A*, *PUM1*, *RPL30*, *RPL37A*, *RPLP0*, *RPS17*, *TBP*, *UBC* and *YWHAZ*) was not significantly different among Day 3, Day 6 and Day 12. Among these genes, *HPRP1* and *RPLP0* were selected as stable reference genes using NormFinder and BestKeeper algorithms.

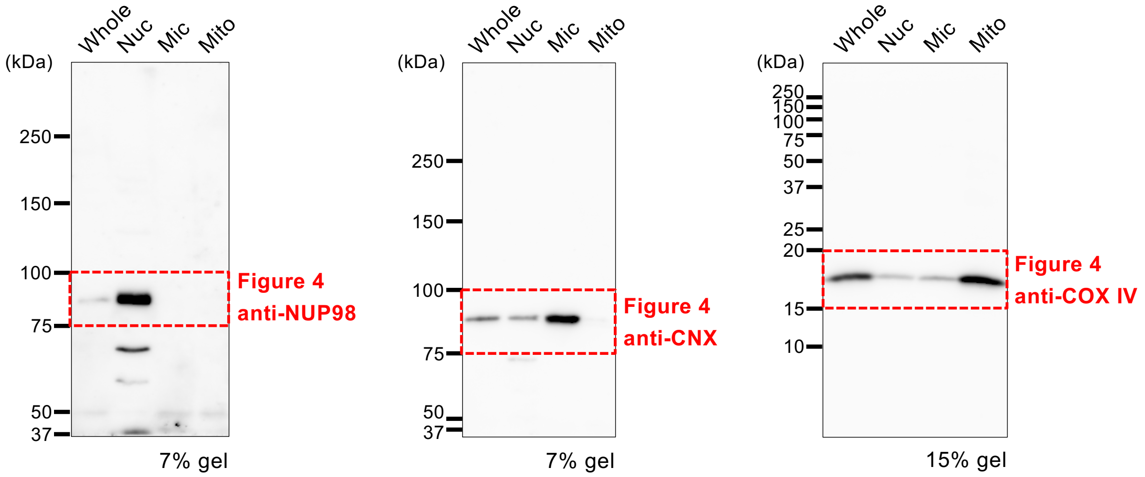

Supplementary Figure S4. Full length blots of Figure 4.
